# Aqueous Extract of *Lonicerae japonicae* Flos Attenuates Alcohol-Associated Liver Disease by Suppressing Oxidative Stress and Inflammation in Mice

**DOI:** 10.3390/ph19101584

**Published:** 2026-10-07

**Authors:** Lin Chen, Mengjie Zhang, Dongsheng Zhao, Xiao Wang, Chuanzhi Kang, Xinyan Qu, Hongjing Dong, Qingjun Li, Jia Li

**Affiliations:** 1 School of Pharmacy, Shandong University of Traditional Chinese Medicine, Jinan 250355, China; mmcl2222@163.com (L.C.); 13561648872@163.com (M.Z.); dongshengpw2008@163.com (D.Z.); 2 Shandong Engineering Research Center for Innovation and Application of General Technology for Separation of Natural Products, Shandong Analysis and Test Center, Qilu University of Technology (Shandong Academy of Sciences), Jinan 250014, China; wangx@sdas.org (X.W.); qxy0117@126.com (X.Q.); donghongjing_2006@163.com (H.D.); 3 State Key Laboratory for Quality Assurance and Sustainable Use of Dao-di Herbs, Institute of Chinese Materia Medica, China Academy of Chinese Medical Sciences, Beijing 100700, China; kangchuanzhi1103@163.com; 4 Experimental Center, Shandong University of Traditional Chinese Medicine, Jinan 250355, China; 5 Key Laboratory of Traditional Chinese Medicine Classical Theory, Ministry of Education, Shandong University of Traditional Chinese Medicine, Jinan 250355, China; 6 Shandong Key Laboratory of Digital Traditional Chinese Medicine, Shandong University of Traditional Chinese Medicine, Jinan 250355, China

**Keywords:** *Lonicerae japonicae* flos, alcohol-associated liver disease, oxidative stress, inflammation, Nrf2/HO-1, p38 MAPK/NF-κB

## Abstract

**Objectives:** This study employed an integrative strategy combining UPLC-MS-based phytochemical profiling, network pharmacology, and transcriptomics to systematically investigate the protective effects and potential mechanisms of LJF against ALD. **Methods:** An ALD model was established via chronic ethanol liquid diet feeding combined with acute ethanol binge administration over 16 days. Liver function, lipid metabolism, oxidative stress (OS), and inflammatory markers were assessed. UHPLC–Q Exactive HFX MS analysis was performed to tentatively annotate the chemical constituents of LJF. Candidate targets and active constituents were predicted by network pharmacology integrated with transcriptomics, followed by experimental validation using quantitative real-time PCR (qRT-PCR) and Western blot. **Results:** LJF extract significantly alleviated ALD, as evidenced by decreased serum aspartate aminotransferase (AST) and alanine aminotransferase (ALT) levels, reduced hepatic total cholesterol (TC), triglyceride (TG), and low-density lipoprotein cholesterol (LDL-C) levels, and improved histopathological changes. LJF attenuated oxidative stress by lowering reactive oxygen species (ROS) and malondialdehyde (MDA) levels while elevating superoxide dismutase (SOD) and glutathione (GSH) levels. Furthermore, LJF suppressed inflammatory responses by reducing IL-6, IL-1β, and TNF-α levels. Integrative analyses pinpointed *Hif1a*, *Ppara*, *Cyp2b10*, *Chac1*, *Nupr1* and *Saa1/2*, among others, as key targets, with luteolin, apigenin, isorhamnetin, lonicerin, 5-O-caffeoyl shikimic acid, and caffeic acid as major bioactive constituents. Mechanistically, LJF concurrently restored the Nrf2/HO-1 antioxidant defense and suppressed the p38 MAPK/NF-κB pro-inflammatory axis, thereby exerting a dual regulatory effect on redox homeostasis and immune response. **Conclusions:** These findings establish LJF as a promising multi-target agent against ALD and, more importantly, provide correlative evidence for the involvement of Nrf2/HO-1 regulation and p38 MAPK/NF-κB signaling modulation, which may guide future strategies for ALD pharmacotherapy.

## 1. Introduction

Alcohol-associated liver disease (ALD) is a major metabolic liver disorder induced by chronic alcohol overconsumption, encompassing a spectrum of pathological changes ranging from simple steatosis to alcohol-associated steatohepatitis (ASH), fibrosis, and ultimately hepatocellular carcinoma (HCC) [1]. With the continuous evolution of modern lifestyles and dietary patterns, the prevalence of heavy alcohol consumption has increased, leading to growing public health concerns related to alcohol misuse and dependence [2]. In accordance with the World Health Organization, approximately 209 million individuals worldwide suffer from alcohol dependence, with alcohol-related deaths reaching 2.6 million annually, accounting for 4.7% of global mortality [3]. Furthermore, clinical guidelines indicate that both alcohol consumption and the prevalence of ALD rise in parallel, underscoring the escalating global burden of this disease [4]. ALD is associated with severe complications, including ascites, variceal bleeding, hepatic encephalopathy, and hepatorenal syndrome, which frequently occur in the cirrhotic stage and contribute substantially to morbidity and mortality [5].

Currently, effective pharmacological therapies for ALD are scarce [6]. The only widely accepted pharmacotherapies for moderate to severe alcohol-associated hepatitis are corticosteroids (e.g., prednisolone), yet their efficacy is modest and inconsistent [7]. Corticosteroids are associated with significant adverse effects, including immunosuppression, hyperglycemia, and gastrointestinal bleeding [8]. Recently, N-acetylcysteine has been used as an adjunct to corticosteroids, but no single agent has proven broadly effective for advanced ALD [9]. In contrast, natural products and herbal extracts have attracted growing attention due to their multi-target characteristics and favorable safety profiles [10]. Given their mild and broad hepatoprotective effects, natural products represent highly promising therapeutic agents. This is particularly true for safe and accessible functional foods and edible plants.

Following absorption, over 90% of ethanol is metabolized in the liver primarily via ADH, CYP2E1, and catalase. Chronic alcohol consumption upregulates CYP2E1 activity, leading to excessive ROS production and oxidative stress [11,12]. To counteract this oxidative insult, cells activate the Nrf2/HO-1 pathway as an endogenous antioxidant defense. However, prolonged alcohol exposure gradually overwhelms or dysregulates Nrf2 signaling, compromising the cellular capacity to scavenge reactive oxygen species (ROS) and exacerbating hepatocellular damage [13]. Concurrently, ROS and the byproducts of alcohol metabolism trigger the activation of mitogen-activated protein kinases (MAPKs) signaling cascades [14]. These byproducts include acetaldehyde and lipid peroxidation intermediates, which activate multiple MAPK signaling cascades, notably the p38 MAPK pathway [15]. Activated p38 MAPK subsequently phosphorylates and activates downstream transcription factors, among which NF-κB is a key driver of alcohol-induced hepatic inflammation [16]. NF-κB activation promotes the transcription of pro-inflammatory cytokines such as TNF-α, IL-6, and IL-1β, which further amplify the inflammatory response and contribute to the progression of ALD [17]. Thus, the interplay between impaired Nrf2-mediated antioxidant defense and sustained NF-κB-driven inflammation constitutes a critical pathological axis in ALD, representing a rational therapeutic target for agents like *Lonicerae japonicae* flos (LJF) that possess both antioxidant and anti-inflammatory properties.

LJF has been used in China to treat febrile diseases, sore throat, and inflammatory conditions such as carbuncles and skin infections. These conditions are traditionally attributed to “heat toxin” in classical Chinese materia medica [18]. Its major bioactive constituents, including chlorogenic acid, luteolin, and isochlorogenic acids, have demonstrated anti-inflammatory, antioxidant, and lipid metabolism-regulatory activities [19,20]. Additionally, LJF extract has been reported to exert notable hepatoprotective effects, highlighting its potential in preventing and treating liver disorders [21,22,23]. LJF has been reported to ameliorate CCl_4_-induced fibrosis [21,24] and APAP-induced liver injury [25], yet its efficacy and mechanistic basis in ALD—particularly regarding the interplay between Nrf2-mediated antioxidant defense and NF-κB-driven inflammation—remain to be determined. ALD is pathologically characterized by inflammation and oxidative stress. These two hallmarks align precisely with the aforementioned pharmacological activities of LJF and its bioactive constituents. We therefore hypothesized that LJF may exert hepatoprotective effects against ALD through coordinated modulation of these pathways.

Therefore, this study aimed to explore the hepatoprotective effects of an aqueous extract of LJF against ALD in a mouse model to investigate whether its mechanism involves the Nrf2-mediated antioxidant defense and NF-κB-driven inflammation via a multi-target mode of action. The pharmacological effects of LJF were systematically assessed in vivo using an ALD model. Network pharmacology integrated with hepatic transcriptomic analysis was subsequently employed to identify possible bioactive components, targets, and signaling pathways. Key pathways were experimentally examined to explore their roles in mediating the hepatoprotective effects of LJF.

## 2. Results

### 2.1. Chemical Analysis of LJF Water Extract

**Figure 1:**
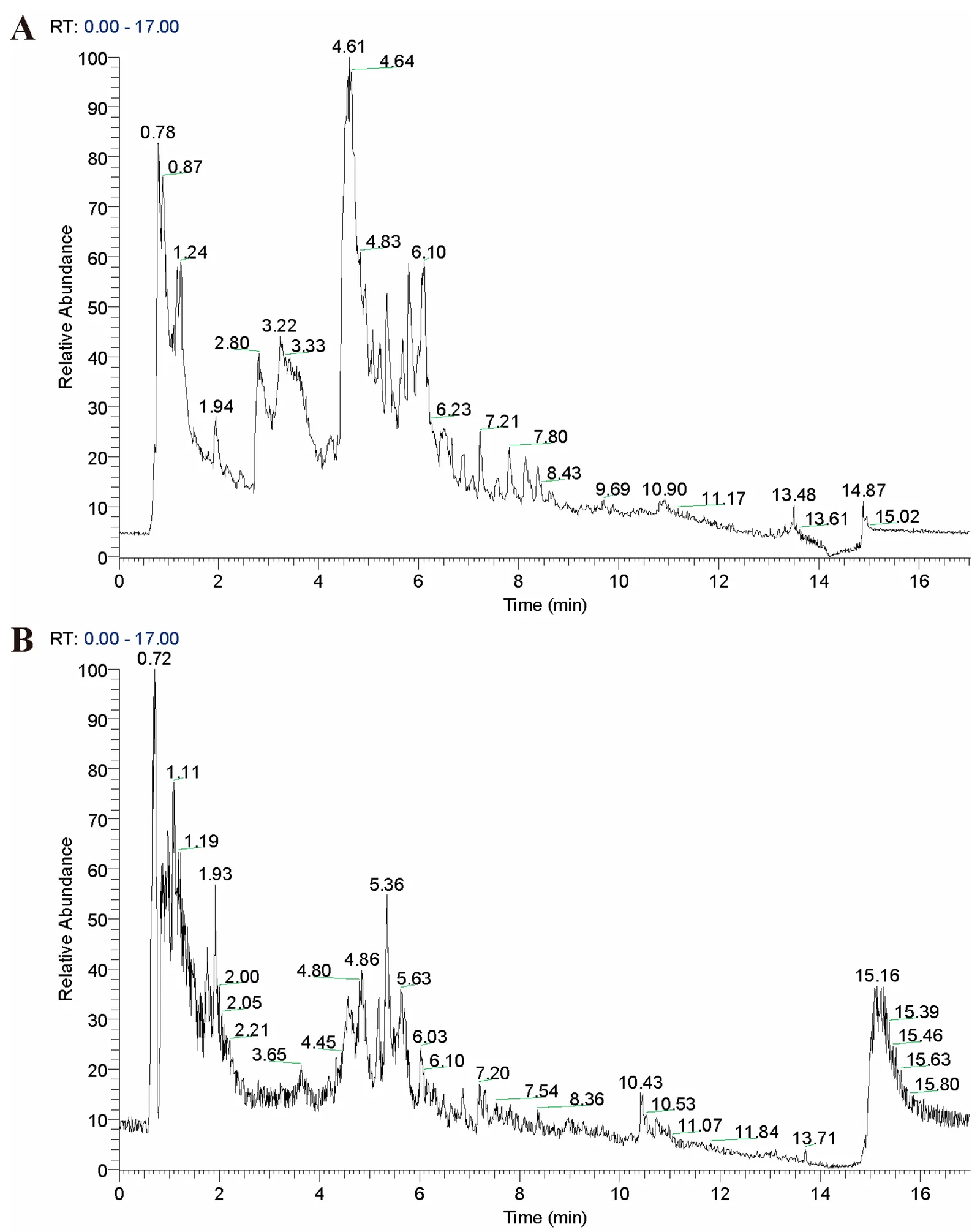
Total ion chromatograms of LJF extract obtained by UHPLC-Q Exactive HFX. (**A**) Negative ion mode. (**B**) Positive ion mode.

Qualitative analysis of the chemical constituents of the LJF water extract was performed using a UHPLC–Q Exactive HFX mass spectrometer in positive and negative ion modes. The total ion chromatograms (TICs) are presented in Figure 1. Compared with the positive ion mode, the negative ion mode exhibited higher total ion current intensity and greater peak abundance, consistent with the major constituents of LJF, such as phenolic acids and flavonoid glycosides, which are more readily ionized in negative ion mode, thereby enhancing detection sensitivity. A total of 40 compounds were tentatively annotated (MSI Level 2) based on accurate mass, retention time, and MS/MS spectral matching against an in-house database, and Table 1 provides the detailed information.

### 2.2. Effects of LJF on Liver Function and Lipid Metabolism in Mice with Alcohol-Associated Liver Disease

**Figure 2:**
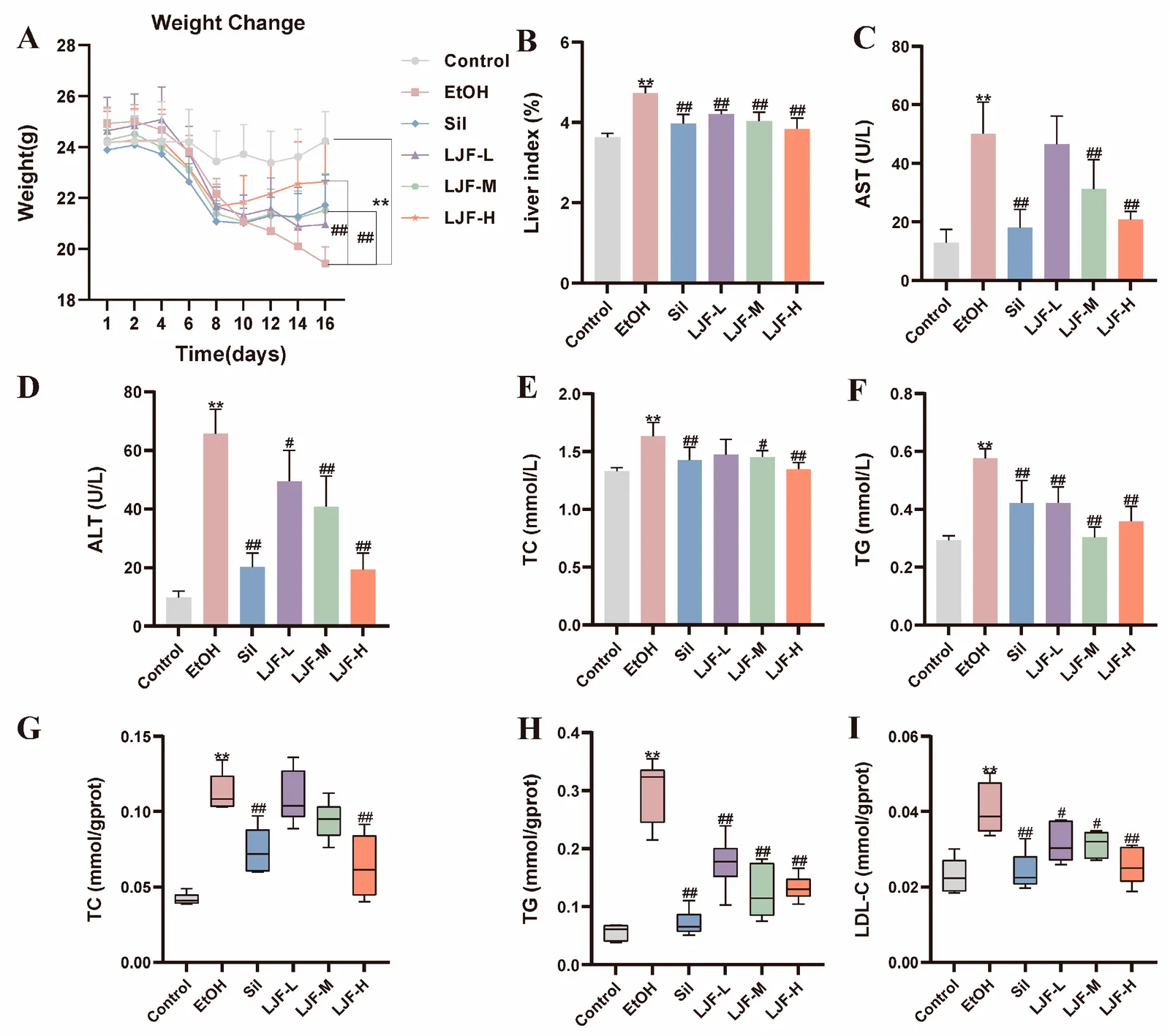
Impacts of LJF upon liver function and lipid metabolism in ALD mice. (*n* = 6) (**A**) Weight change. (**B**) Liver index. (**C**) Serum AST levels. (**D**) Serum ALT levels. (**E**) Serum TC levels. (**F**) Serum TG levels. (**G**) Hepatic TC levels. (**H**) Hepatic TG levels. (**I**) Hepatic LDL-C levels. Compared with the control group, ** *p* < 0.01; compared with the EtOH group, ^#^ *p* < 0.05, ^##^ *p* < 0.01.

As shown in Figure 2A, body weight in each group exhibited distinct trends over the 16-day experimental period. Mice in the control group showed a steady and modest increase in body weight. In contrast, mice in the EtOH group displayed a marked decline, starting from day 6, which persisted until day 16, indicating that chronic alcohol exposure induced significant weight loss. Compared with the EtOH group, mice treated with low-, medium-, and high-dose LJF exhibited attenuated weight loss, with body weight gradually stabilizing and partially recovering during the later stages, suggesting a protective effect of LJF against alcohol-induced weight loss.

The liver index (Figure 2B), a direct indicator of liver injury, was significantly elevated in the EtOH group compared with controls (*p* < 0.01). LJF treatment significantly decreased the liver index in a dose-dependent manner (*p* < 0.01). Serum AST and ALT levels, classical biomarkers of hepatocellular injury, were markedly increased in the EtOH group relative to controls (*p* < 0.01), confirming successful induction of the ALD model (Figure 2C,D). Treatment with LJF aqueous extract significantly reduced serum AST and ALT levels in a dose-dependent manner, with the high-dose group showing the most pronounced effect (*p* < 0.01). These results indicate that LJF effectively ameliorates ALD, preserves hepatocyte membrane integrity, and improves liver function.

In addition, mice in the EtOH group exhibited significantly elevated levels of TC and TG in both serum (Figure 2E,F) and liver (Figure 2G,H), along with increased hepatic LDL-C levels (Figure 2I), compared with controls (*p* < 0.01), indicating disrupted lipid metabolism induced by alcohol exposure. Treatment with silybin or LJF aqueous extract significantly decreased TC, TG, and LDL-C levels to varying degrees (*p* < 0.05 or *p* < 0.01), with the high-dose LJF group demonstrating the greatest improvement.

### 2.3. Effects of LJF on Oxidative Stress and Inflammatory Response in Mice with Alcohol-Associated Liver Disease

**Figure 3:**
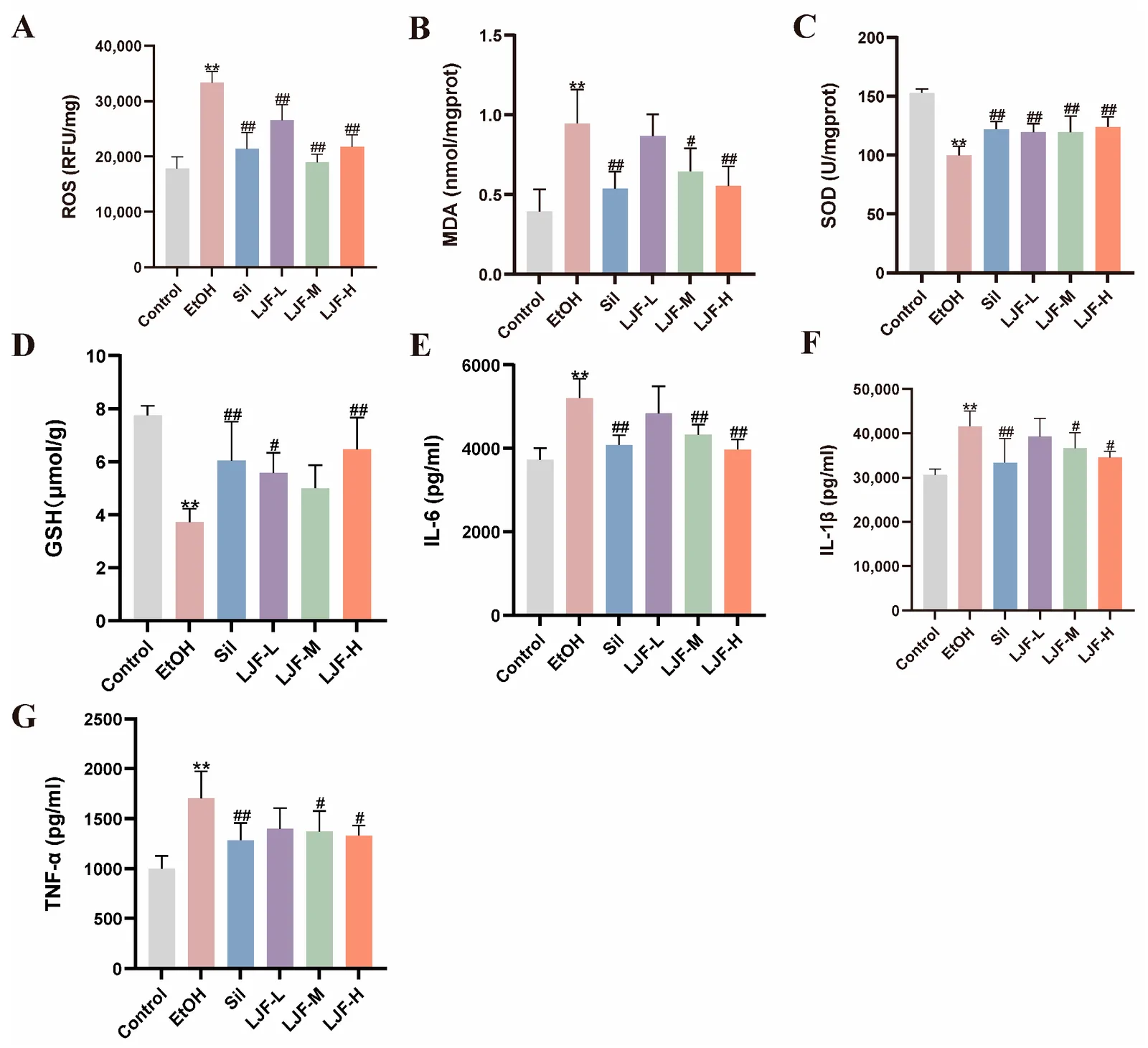
Effects of LJF on oxidative stress and inflammation in mice with ALD. (*n* = 6) (**A**) Hepatic ROS levels. (**B**) Hepatic MDA levels. (**C**) Hepatic SOD activity. (**D**) Hepatic GSH levels. (**E**) Hepatic IL-6 levels. (**F**) Hepatic IL-1β levels. (**G**) Hepatic TNF-α levels. Compared with the control group, ** *p* < 0.01; compared with the EtOH group, ^#^ *p* < 0.05, ^##^ *p* < 0.01.

Compared with controls, mice in the EtOH group displayed significantly elevated levels of ROS (Figure 3A) and MDA (Figure 3B) in liver tissues (*p* < 0.01), accompanied by markedly decreased SOD activity (Figure 3C) and GSH content (Figure 3D) (*p* < 0.01). This indicates that alcohol intake triggers oxidative stress and impairs the endogenous antioxidant defense system. Compared with the EtOH group, all LJF-treated groups displayed varying degrees of reduction in hepatic ROS and MDA levels, along with a significant increase in SOD activity and GSH content (*p* < 0.01 or *p* < 0.05). Thus, LJF alleviates alcohol-induced oxidative stress by inhibiting lipid peroxidation and enhancing antioxidant enzyme activities, thereby exerting hepatoprotective effects.

Hepatic levels of pro-inflammatory cytokines IL-6, IL-1β, and TNF-α were measured by ELISA (Figure 3E–G). Compared with controls, mice in the EtOH group showed significantly elevated hepatic levels of these cytokines (*p* < 0.01), indicating that alcohol exposure successfully induced a hepatic inflammatory response. Compared with the EtOH group, LJF treatment at all doses decreased the levels of these cytokines to various degrees, showing a dose-dependent trend. Notably, the medium- and high-dose LJF groups significantly reduced all three cytokines (*p* < 0.05 or *p* < 0.01). These results indicate that LJF suppresses alcohol-induced inflammatory responses by inhibiting pro-inflammatory cytokine production in the liver, thereby contributing to its hepatoprotective effects.

### 2.4. Effects of LJF on Hepatic Histopathology in Mice with Alcohol-Associated Liver Disease

**Figure 4:**
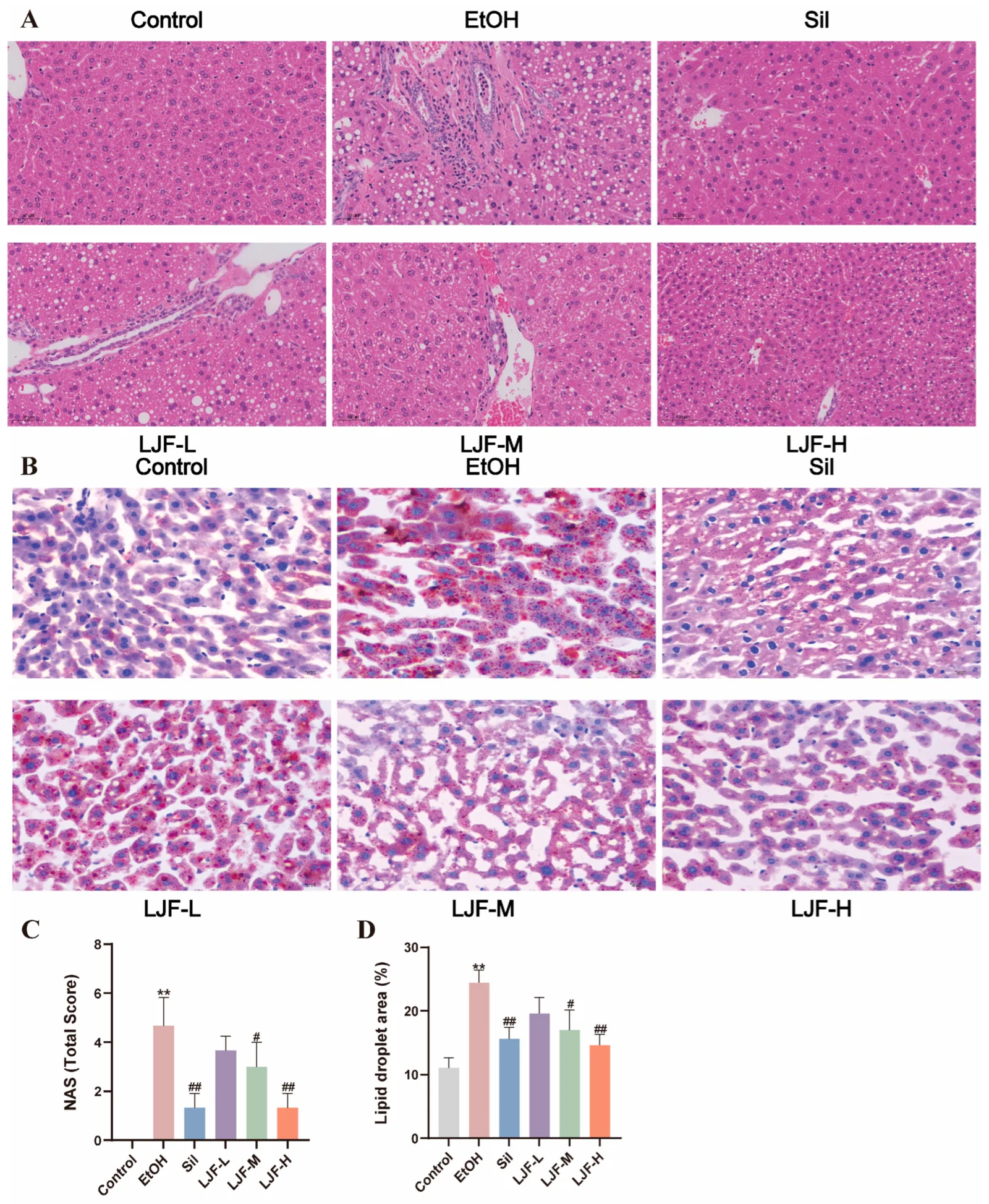
Impacts of LJF on hepatic histopathology in mice with ALD. (*n* = 3) (**A**) Representative images of liver histopathology (H&E staining). Bar = 50 µm. (**B**) Representative images of Oil Red O staining of liver sections. Bar = 10 µm. (**C**)NAS (Total Score). (**D**) Lipid droplet area (%). Compared with the control group, ** *p* < 0.01; compared with the EtOH group, ^#^ *p* < 0.05, ^##^ *p* < 0.01.

As shown in Figure 4A, HE staining revealed that control mice exhibited a well-organized and intact liver architecture, with hepatocytes showing normal morphology, round centrally located nuclei, and uniform cell size. In the EtOH group, hepatocytes displayed marked cytoplasmic vacuolization along with inflammatory cell infiltration. Compared with the EtOH group, these pathological alterations were visibly alleviated in the silybin group and all LJF-treated groups. Hepatic steatosis and cytoplasmic vacuolization were markedly reduced, and inflammatory cell infiltration was attenuated, indicating that LJF effectively alleviated alcohol-induced hepatic histopathological damage.

Oil Red O staining was performed to assess the effects of LJF on hepatic lipid accumulation in mice with ALD (Figure 4B). Liver sections from controls exhibited almost no Oil Red O-positive staining, indicating minimal intracellular lipid deposition and normal lipid metabolism. In contrast, extensive orange-red staining with numerous lipid droplets was observed in the EtOH group, suggesting severe lipid accumulation induced by alcohol exposure. Compared with the EtOH group, treatment with silybin or LJF at all dosages visibly reduced Oil Red O-positive areas, indicating an amelioration of hepatic lipid accumulation. Notably, the high-dose LJF group showed a staining pattern visually similar to that of the normal control group.

### 2.5. Network Pharmacology Analysis

**Figure 5:**
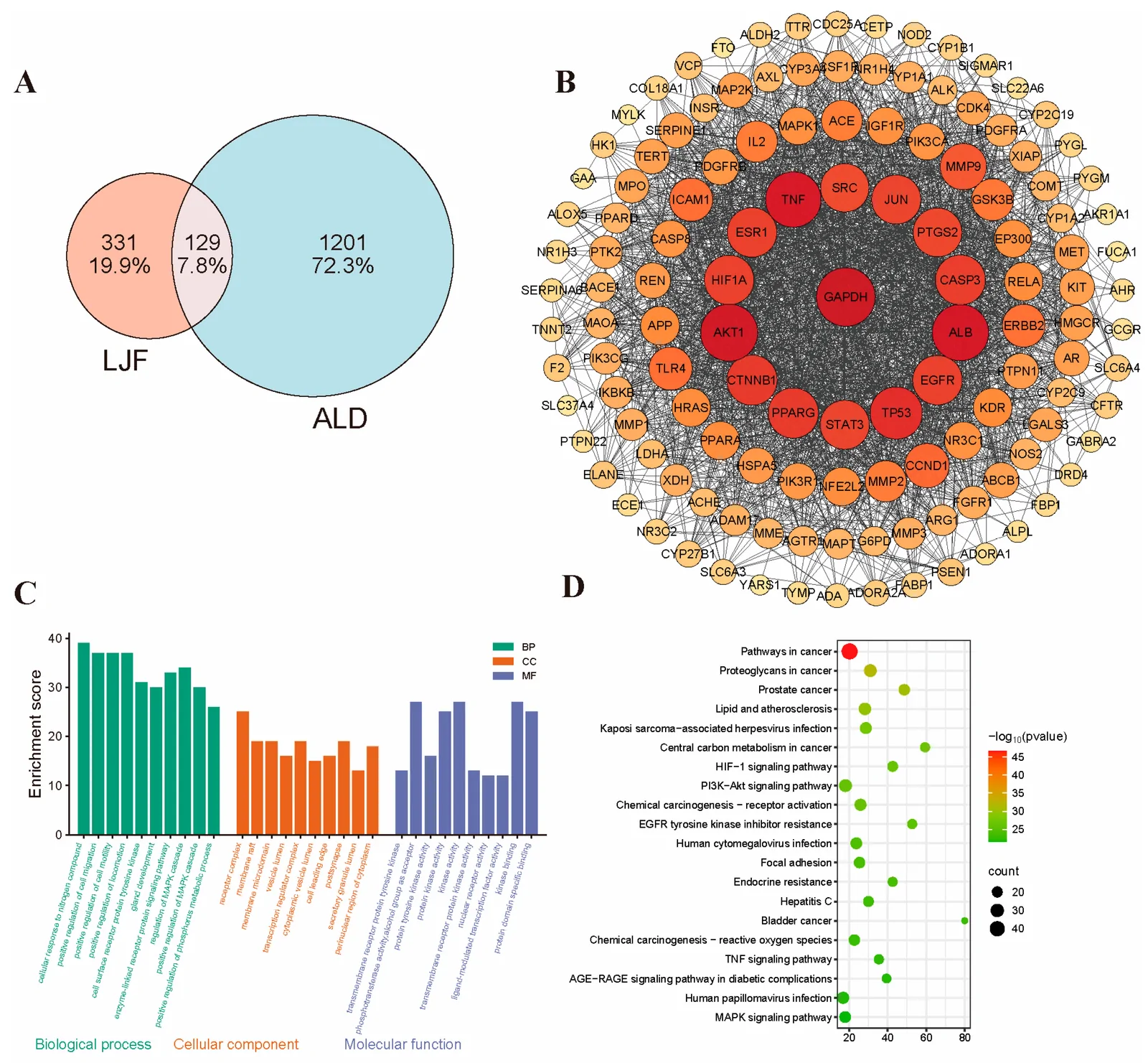
Network Pharmacology Analysis. (**A**) Intersection analysis of LJF ingredient-putative targets and ALD-putative targets. (**B**) PPI network of potential targets, with node size indicating degree value; larger nodes represent higher degree values. (**C**) The diagram of GO enrichment analysis for LJF genes. (**D**) The diagram of KEGG enrichment analysis for LJF genes based on the Metascape database. The top 20 KEGG pathways are shown.

Candidate bioactive compounds of LJF were screened by integrating TCMSP database screening with mass spectrometry data, further supplemented with major active constituents reported in the literature [26,27,28], yielding a total of 66 compounds. The underlying targets of these compounds were predicted using the Swiss Target Prediction database, resulting in 460 non-redundant targets after duplicate removal. Meanwhile, 1330 ALD-related targets were retrieved from public databases, and intersection analysis identified 129 potential therapeutic targets (Figure 5A).

These intersecting targets were submitted to the STRING database to construct a PPI network, which contained 129 nodes and 2126 edges, representing target proteins and their interactions, respectively. The network was visualized and analyzed using Cytoscape 3.7.1 (Figure 5B). Topological analysis was based on parameters such as degree centrality (DC), betweenness centrality (BC), closeness centrality (CC), eigenvector centrality (EC), local average connectivity (LAC), and network score ultimately identified 11 core targets: *Tnf, Akt1, Hif1a, Pparg, Ctnnb1, Ppara, Nfe2l2, Ptgs2, Stat3, Casp3,* and *Jun*. Combined with degree values and MS/MS relative abundance, the key bioactive compounds were determined as luteolin, apigenin, isorhamnetin, lonicerin, 5-O-caffeoyl shikimic acid, caffeic acid, and ursolic acid.

Gene Ontology (GO) enrichment analysis was performed using the Metascape platform, and the top 10 terms ranked by *p*-value were chosen in Figure 5C. In the biological process (BP) category, the core targets were mainly enriched in signal transduction and metabolic regulation. In the molecular function (MF) category, they were closely associated with phosphotransferase activity, transcription factor activity, and protein kinase activity. These results suggest that LJF may exert therapeutic effects on ALD by regulating intracellular signaling, gene transcription, and protein phosphorylation.

For KEGG pathway enrichment, a threshold of *p* < 0.01 was applied, and the top 20 significantly enriched pathways were visualized (Figure 5D). Obviously, the core targets were significantly enriched in pathways related to cell survival, inflammation, and metabolism. The enrichment of the PI3K-Akt and MAPK pathways was consistent with the GO findings on protein kinase activity and signal transduction, suggesting that LJF may promote hepatocyte survival and repair through activation of these cascades. In addition, significant enrichment in the TNF and HIF-1 signaling pathways suggests that LJF may alleviate ALD by regulating oxidative stress and inflammation. Collectively, the KEGG analysis indicates that LJF exerts multi-target hepatoprotective effects against ALD via coordinated regulation of pathways involved in cell survival, inflammation, and oxidative stress.

### 2.6. Transcriptomic Analysis of the Liver

**Figure 6:**
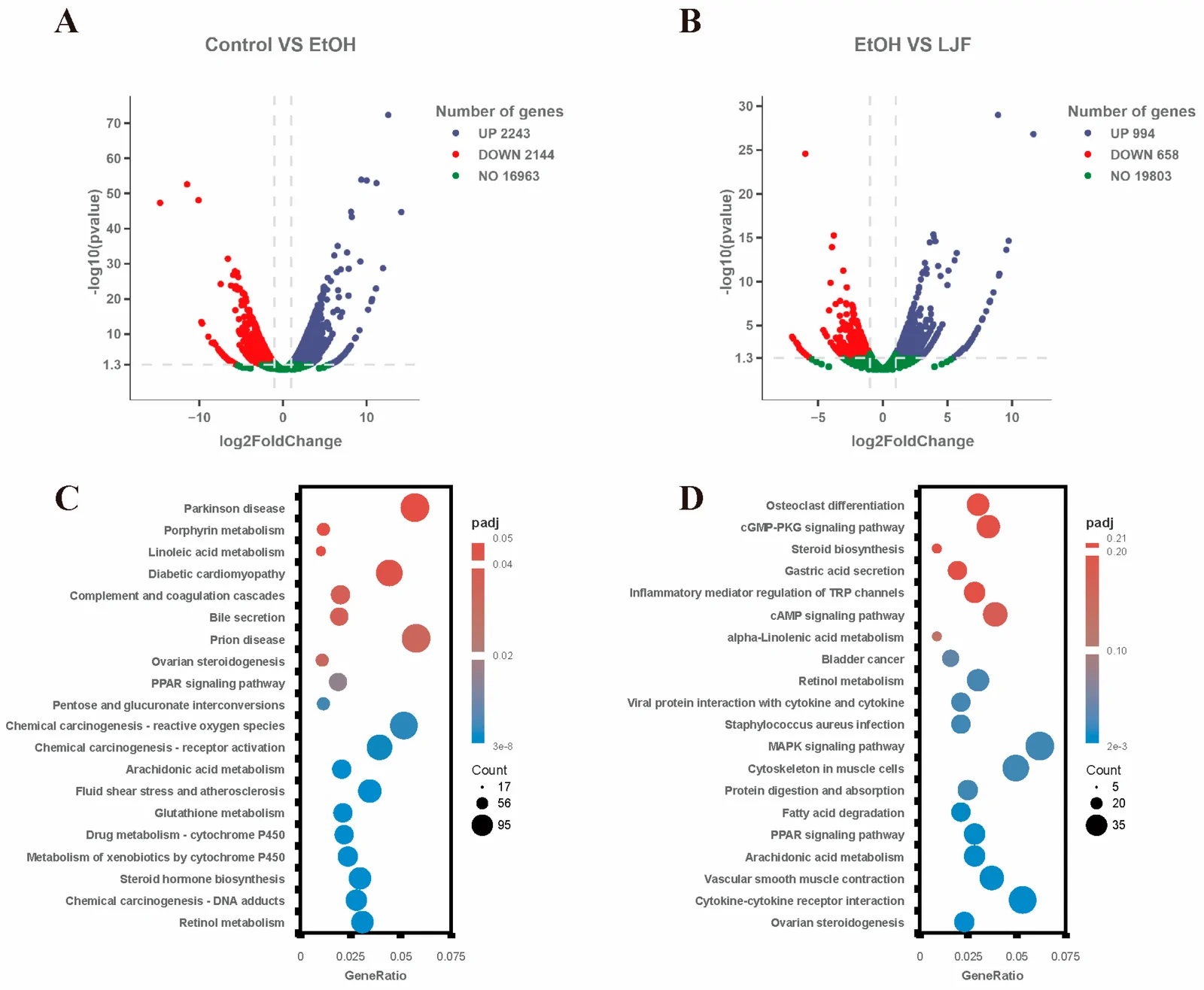
Transcriptomic Analysis of the Liver. (**A**) Volcano Plot of DEGs between Control and EtOH Groups. (**B**) Volcano Plot of DEGs between EtOH and LJF Groups. (**C**) KEGG pathway enrichment analysis between Control and EtOH groups. (**D**) KEGG pathway enrichment analysis between EtOH and LJF Groups.

To investigate gene expression alterations associated with ALD, differentially expressed genes (DEGs) were identified using the thresholds of |log2(fold change)| ≥ 1 and adjusted *p*-value ≤ 0.05. A total of 4387 DEGs were identified between the Control and EtOH groups, including 2144 downregulated and 2243 upregulated genes (Figure 6A). In addition, 1652 DEGs were identified between the EtOH and LJF groups, comprising 658 downregulated and 994 upregulated genes (Figure 6B). Among these, 3517 DEGs were unique to the Control vs. EtOH comparison, 782 were unique to the EtOH vs. LJF comparison, and 870 genes were shared between both comparisons. Based on the enrichment analysis of overlapping genes, several genes, including *Cyp2b10, Gadd45a, Hmox1, Atf3, Chac1, Nupr1, Trib3, Lcn2, Ddit3, Gdf15, Myc, Hsd3b5*, and *Saa1/2* were identified as potential targets of LJF in treating ALD.

KEGG pathway enrichment analysis of the Control vs. EtOH comparison revealed the key biological processes associated with ALD (Figure 6C). These processes can be broadly categorized into metabolic dysregulation, lipid metabolism disorder, and oxidative stress imbalance. The cytochrome P450 enzyme system, a central component of hepatic metabolism, was significantly enriched in pathways such as drug metabolism-cytochrome P450 and metabolism of xenobiotics by cytochrome P450, indicating its involvement in alcohol-induced toxic metabolism. Regarding antioxidant defense, enrichment of pathways including glutathione metabolism and arachidonic acid metabolism suggests impaired antioxidant capacity and enhanced oxidative stress. Furthermore, pathways related to lipid metabolism and inflammatory regulation, particularly the PPAR signaling pathway, were significantly altered under alcohol exposure, potentially contributing to disrupted lipid homeostasis, bile acid metabolism imbalance, and enhanced inflammatory responses. KEGG enrichment analysis of the EtOH vs. LJF comparison indicated that the protective effects of LJF against ALD are mainly associated with the PPAR and the MAPK signaling pathways (Figure 6D).

### 2.7. Effects of LJF on the Nrf2/HO-1 Signaling Pathways in Mice with Alcohol-Associated Liver Disease

**Figure 7:**
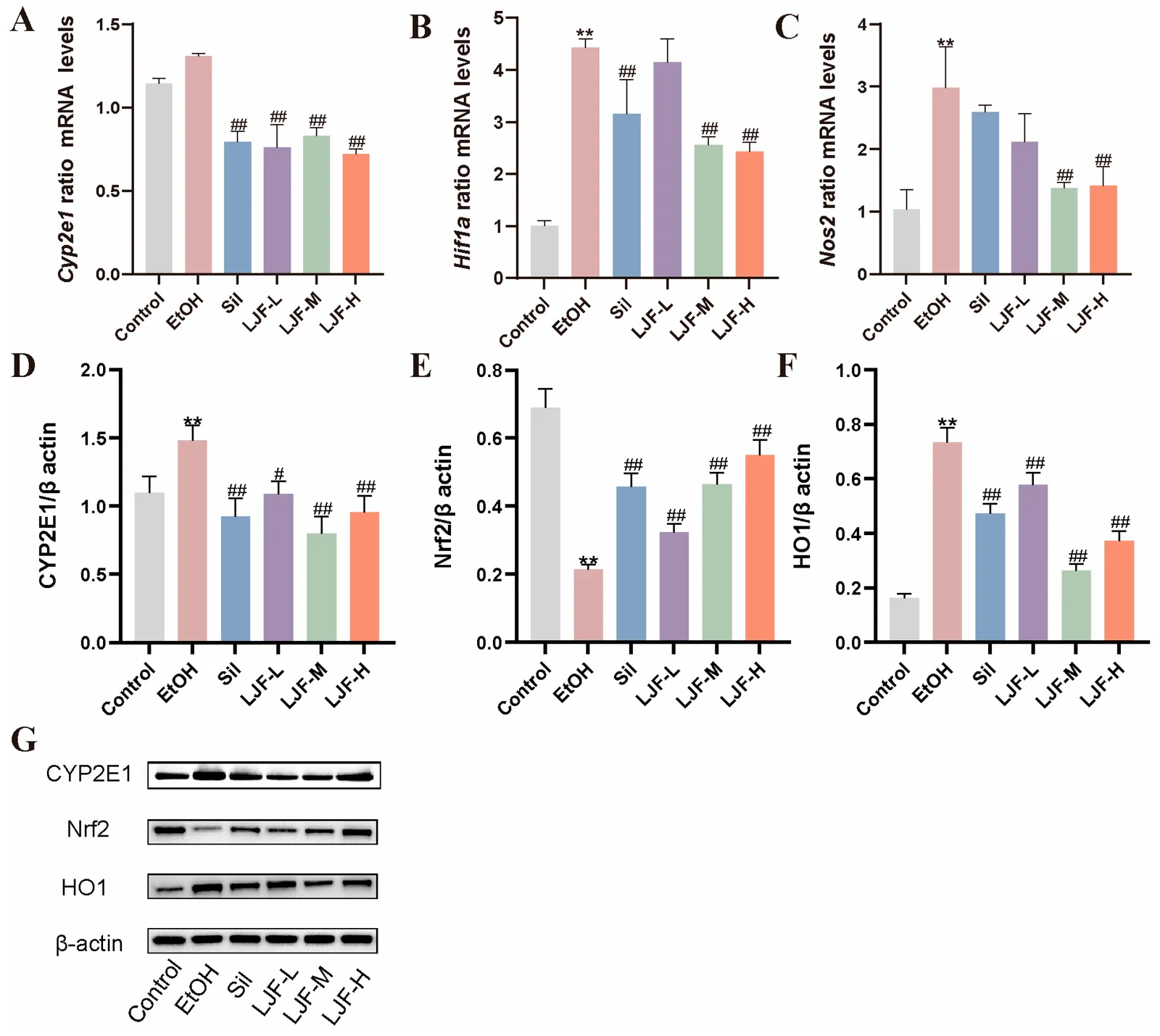
Effects of LJF upon the Nrf2/HO-1 signaling pathways among mice with ALD. (*n* = 3) (**A**–**C**) mRNA expression of *Cyp2e1, Hif1a* and *Nos2* in the liver. (**D**–**F**) Quantification of CYP2E1, Nrf2 and HO1 protein expression. (**G**) CYP2E1, Nrf2 and HO1 expression in the liver determined by Western blot. Compared with the control group, ** *p* < 0.01; compared with the EtOH group, ^#^ *p* < 0.05, ^##^ *p* < 0.01.

Regarding oxidative stress-related genes, mice in the EtOH group showed significantly elevated hepatic *Cyp2e1* mRNA levels (Figure 7A) in contrast to controls, indicating a dysregulation of this key ROS-generating enzyme during alcohol metabolism. LJF treatment significantly downregulated *Cyp2e1* expression to varying degrees (*p* < 0.05 or *p* < 0.01), suggesting that LJF may alleviate oxidative stress by modulating the expression of key pro-oxidant genes. Additionally, the mRNA expression levels of *Hif1a* (Figure 7B) and *Nos2* (Figure 7C) were significantly increased in the EtOH group (*p* < 0.01), further indicating enhanced pro-oxidant gene expression induced by alcohol exposure. CYP2E1 is an essential enzyme responsible for ROS generation during alcohol metabolism, while Nrf2 serves as a central transcription factor regulating cellular antioxidant responses, and its downstream target HO-1 plays an essential role in free radical scavenging and maintenance of redox homeostasis. We therefore assessed the protein levels of these targets by Western blot. Compared with controls, CYP2E1 protein expression (Figure 7D) was significantly elevated in the EtOH group and was markedly reduced following LJF treatment. Total Nrf2 protein expression (Figure 7E) was markedly decreased in the EtOH group (*p* < 0.01). In contrast, HO-1 protein expression (Figure 7F) was significantly increased, which may reflect a compensatory antioxidant response under alcohol exposure. This seemingly paradoxical increase in HO-1, despite reduced total Nrf2, may reflect a compensatory feedback loop driven by sustained oxidative stress, possibly involving redistribution or altered turnover of Nrf2 protein, rather than a direct consumption event. Treatment with LJF aqueous extract restored total Nrf2 protein expression in a dose-dependent manner, with levels in the high-dose group approaching those of the controls. Concurrently, HO-1 protein expression was significantly reduced in all treatment groups (*p* < 0.01). Collectively, these results suggest that LJF alleviates oxidative stress and is associated with changes in the Nrf2/HO-1 pathway. However, direct evidence for Nrf2 activation (e.g., nuclear translocation or transcriptional activity) was not obtained in this study, and further investigation is warranted.

### 2.8. Effects of LJF on the p38 MAPK/NF-κB Signaling Pathways in Mice with Alcohol-Associated Liver Disease

**Figure 8:**
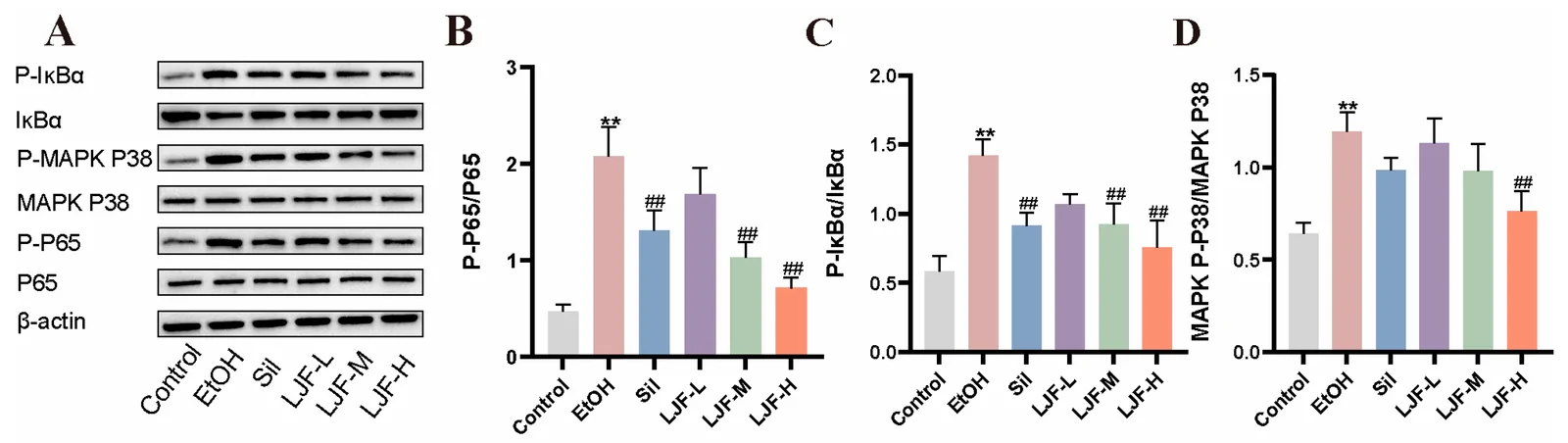
Effects of LJF upon the p38 MAPK/NF-κB signaling pathways in mice with ALD. (*n* = 3) (**A**) p-IκBα, IκBα, MAPK p-p38, MAPK p38, NF-κB p65 and p-NF-κB p65 expression in the liver determined by Western blot. (**B**) Quantification of p-NF-κB p65 relative to total NF-κB p65. (**C**) Quantification of p-IκBα relative to total IκBα. (**D**) Quantification of MAPK p-p38 relative to total p38. For phosphorylated proteins, both phosphorylated and total protein bands were first normalized to β-actin, and the phosphorylation level is presented as the phospho/total ratio. Compared with the control group, ** *p* < 0.01; compared with the EtOH group, ^##^ *p* < 0.01.

Compared with the Control group, the hepatic p-NF-κB p65/total p65 ratio (Figure 8B) was markedly increased in the EtOH group (*p* < 0.01). Similarly, the p-IκBα/total IκBα ratio (Figure 8C) was markedly elevated in the EtOH group, indicating enhanced IκBα phosphorylation. After treatment with LJF aqueous extract, the p-NF-κB p65/total p65 ratio was significantly reduced in a dose-dependent manner (*p* < 0.05 or *p* < 0.01), with the high-dose group showing the most pronounced effect. In addition, the p-IκBα/total IκBα ratio was significantly decreased by LJF treatment, and the β-actin-normalized total IκBα level was restored to a level comparable to that of the Control group. To further investigate the upstream regulatory mechanism of NF-κB activation, the phosphorylation status of MAPK p38 was examined. The p-p38/total p38 ratio (Figure 8D) was markedly increased in the EtOH group compared with the Control group (*p* < 0.01). In contrast, LJF treatment markedly reduced this ratio, suggesting that LJF may suppress NF-κB activation through inhibition of p38 MAPK phosphorylation.

## 3. Discussion

Excessive alcohol consumption induces multisystem damage, affecting the central nervous system and key organs such as the liver, heart, and brain through mechanisms involving oxidative stress, lipid accumulation, and inflammation. Among these, ALD is a major health concern worldwide. Therefore, there is an urgent need to develop safe and effective functional foods and pharmacological agents that can enhance ethanol metabolism and protect liver function. Given the limitations of single-target therapies, growing attention has been directed toward natural products with multi-target regulatory properties. As a representative “medicine-food homology” herb, LJF has demonstrated hepatoprotective potential, largely attributed to its antioxidant and anti-inflammatory activities [24,29,30].

ALD pathogenesis is driven by multiple interrelated factors, including ethanol toxicity, lipid metabolism disorders, inflammation, and oxidative stress. After ingestion, ethanol is metabolized in hepatocytes to acetaldehyde and then to acetate [31]. Under excessive alcohol exposure, CYP2E1 becomes a major metabolic enzyme [32], e generating large amounts of ROS and consuming NADPH, thereby impairing GSH regeneration and disrupting redox homeostasis [33,34]. This oxidative imbalance promotes lipid peroxidation and mitochondrial dysfunction, which in turn triggers inflammatory responses [35,36].

Consistent with the pathological features of ALD, the EtOH group displayed marked body weight loss, elevated liver index, increased serum ALT and AST levels, and pronounced histopathological damage, confirming successful model establishment. LJF treatment markedly ameliorated these alterations, demonstrating hepatoprotective effects. Hepatic steatosis serves as an early and critical event in ALD development [37], arising from ethanol-induced disruption of fatty acid oxidation and promotion of triglyceride accumulation in hepatocytes [38,39]. In this study, LJF markedly reduced serum and hepatic TC, TG, and LDL-C levels and alleviated lipid accumulation, as confirmed by Oil Red O staining. PPARα, an important nuclear transcription factor modulating fatty acid oxidation, is essential for maintaining hepatic lipid homeostasis [40]. Chronic ethanol intake suppresses PPARα expression and activity, impairing β-oxidation and promoting lipid accumulation. Conversely, SREBF1 regulates genes involved in fatty acid synthesis, and its overactivation drives alcoholic fatty liver development [41]. Ethanol exposure upregulates SREBF1 and downstream targets such as FASN and ACC1 [42], further exacerbating lipid accumulation. The downregulation of PPARα combined with upregulation of SREBF1 may synergistically promote fatty acid synthesis while suppressing fatty acid oxidation, thus exacerbating hepatic lipid accumulation [43]. Importantly, excessive lipid accumulation resulting from dysregulated lipid metabolism further contributes to oxidative stress through enhanced lipid peroxidation and ROS generation.

Chronic excessive alcohol intake upregulates CYP2E1 expression in the hepatic endoplasmic reticulum, disrupts mitochondrial respiration, and generates excessive ROS, leading to cellular injury [44]. To counteract this oxidative burden, endogenous antioxidants such as SOD and GSH are consumed to maintain redox homeostasis. SOD eliminates superoxide anions, terminating lipid peroxidation chain reactions [45], while GSH directly scavenges free radicals and protects against ROS-mediated toxicity [46]. When antioxidant defenses are insufficient, lipid peroxidation occurs, with MDA serving as a key biomarker [47]. In this study, LJF aqueous extract significantly decreased hepatic ROS and MDA levels, restored GSH and SOD, enhanced antioxidant capacity, and alleviated ethanol-induced oxidative stress. Persistent oxidative stress further triggers inflammatory responses, as evidenced by elevated IL-6, IL-1β, and TNF-α. Notably, LJF treatment significantly inhibited these pro-inflammatory cytokines, indicating that its hepatoprotective effects are partly attributable to anti-inflammatory mechanisms.

HIF1A, a central transcription factor in hypoxia and oxidative stress responses, is upregulated by ethanol and contributes to lipid metabolic disturbances, inflammation, apoptosis, and fibrosis [48,49]. iNOS, another key enzyme in ethanol-induced oxidative and nitrosative stress, produces NO, which reacts with superoxide to form peroxynitrite (ONOO−), damaging cellular proteins and impairing mitochondrial function [50,51]. HIF1A directly regulates iNOS under hypoxia, further exacerbating oxidative injury. In this study, LJF treatment downregulated both *Hif1a* and *Nos2*, suggesting a potential improvement in hepatic redox status. A particularly intriguing finding is the paradoxical uncoupling of total Nrf2 protein expression from its downstream effector HO-1. Although total hepatic Nrf2 was significantly decreased in the EtOH group, HO-1 expression was unexpectedly elevated. This seemingly contradictory observation does not necessarily indicate Nrf2-independent HO-1 regulation; rather, it may reflect a compensatory feedback response driven by sustained oxidative stress. Under chronic alcohol exposure, one possibility is that residual Nrf2 undergoes enhanced nuclear translocation and transcriptional activity, accompanied by accelerated cytoplasmic Nrf2 degradation via the ubiquitin-proteasome pathway [52,53]; however, this remains speculative because these processes were not directly assessed in the present study. Thus, the net effect observed was reduced total Nrf2 but upregulated HO-1, which might represent a maladaptive response rather than a purely protective one. LJF treatment restored total Nrf2 protein expression and reduced HO-1 expression to levels comparable to those of the Control group, suggesting that its effects may be associated with the Nrf2/HO-1 pathway rather than a purely direct antioxidative action. However, since Nrf2 nuclear translocation and transcriptional activity were not directly assessed, this interpretation should be considered tentative.

Inflammation is a critical driver in the development of ALD from simple steatosis to steatohepatitis and fibrosis [54]. ROS generated during ethanol metabolism, together with endotoxins from gut barrier disruption, synergistically activate hepatic inflammatory signaling cascades [55]. LPS binds to TLR4 on cell membrane, recruiting downstream adaptor proteins such as MD-2 and MyD88, which trigger intracellular signaling and inflammation [56,57]. The inflammatory cascade in ALD is critically governed by the p38 MAPK/NF-κB axis, yet the interplay between oxidative stress and this pathway in the context of LJF intervention remains poorly defined. Our results show that LJF concurrently suppressed p38 phosphorylation, IκBα phosphorylation, and NF-κB p65 phosphorylation. Notably, the inhibition of p38 MAPK activation preceded or paralleled the reduction in pro-inflammatory cytokines, suggesting that p38 may serve as an upstream integrator of both oxidative and inflammatory signals in this model. This is particularly relevant because p38 MAPK directly phosphorylates the p65 subunit and also facilitates IKK-mediated IκBα degradation [58]. The simultaneous reduction in phosphorylation at these signaling nodes by LJF is not consistent with a single-target mechanism and instead suggests a multi-tiered modulatory effect on the MAPK/NF-κB cascade, potentially involving upstream redox-sensitive kinases.

Despite these promising findings, several limitations should be acknowledged. First, although 10 mice were initially assigned to each group, the sample sizes for histology, Western blotting, and transcriptomic analyses were relatively small (n = 3 per group), and blinding was applied only to histological scoring; these endpoints should therefore be considered exploratory or supportive rather than definitive. Second, we measured total protein levels but did not assess subcellular translocation or DNA-binding activities, which would strengthen functional evidence for the Nrf2/HO-1 pathway. Future work should validate these findings in additional ALD models and with larger sample sizes and blinded assessment, and should include bioactivity-guided fractionation and pharmacological inhibitor studies to clarify pathway hierarchies. Nevertheless, our findings support LJF as a multi-target agent that may restore lipid metabolism, redox balance, and inflammatory signaling in ALD.

## 4. Materials and Methods

### 4.1. Preparation of Aqueous Extract of LJF

The dried flower buds of Lonicera japonica Thunb. were purchased from Shandong Zhongping Pharmaceutical Co., Ltd. (Pingyi, Shandong, China) (batch No. 20240803), with the geographical origin confirmed as Pingyi County, Shandong Province, China. The botanical identity was authenticated by Prof. Jia Li at Shandong University of Traditional Chinese Medicine, based on morphological characteristics described in the Flora of China. The raw materials were stored in a sealed container, protected from light, at 20–25 °C with a relative humidity of ≤60% until extraction. Dried LJF (300 g) was soaked in distilled water at a ratio of 1:10 (*w*/*v*) for 1 h, and then reflux-extracted at 100 °C for 1 h. The extract was filtered through four layers of gauze and the residue was re-extracted with distilled water at a ratio of 1:8 (*w*/*v*) under the same conditions. The combined filtrates were concentrated under reduced pressure using a rotary evaporator at 65 °C to a final volume of 272.5 mL [59], and the resulting concentrate was freeze-dried to yield 46.46% (*w*/*w*) powder.

### 4.2. Chemical Composition Analysis of the Aqueous Extract of LJF

The freeze-dried LJF powder was extracted with isopropanol/acetonitrile/water by vortexing and ultrasonication at 4 °C. After centrifugation, the supernatant was evaporated to dryness under vacuum and reconstituted with 200 µL of 50% acetonitrile. The resulting solution was centrifuged to remove insoluble debris. Samples were analyzed via UPLC-Orbitrap-MS (Vanquish-HFX, Thermo Fisher Scientific, Germany) with a Waters HSS T3 column (100 × 2.1 mm, 1.8 µm, 40 °C), 0.3 mL/min flow, 2 µL injection. Mobile phase A = water + 0.1% formic acid; B = acetonitrile + 0.1% formic acid. Gradient: 100% A for 1 min, to 5% A over 11 min, held for 1 min, back to 100% A at 13.1 min, equilibrated to 17 min. MS: Q Exactive HFX Orbitrap (heated ESI, Thermo Fisher Scientific, Bremen, Germany) in Full MS/dd-MS2; ESI settings: sheath/aux gas 40/10 arb, spray voltage ±3000/2800 V, source 350 °C, ion tube 320 °C; full scan *m*/*z* 70–1050 at 70 k, MS/MS at 17.5 k [60].

Raw data were processed using Progenesis QI (version 3.0, Waters Corporation, Milford, CT, USA) for baseline filtering, peak detection, alignment, and retention-time correction, generating a data matrix of retention time, *m*/*z*, and peak intensity. Compound annotation was performed against a custom in-house MS/MS database of traditional Chinese medicinal herbs, with fragment matching assessed by MS2 scores (ranging from 0 to 1, with scores > 0.5 considered reliable).

Compounds were tentatively annotated based on matching with the Sanshu Biotechnology in-house traditional Chinese medicine database, mass accuracy within 10 ppm, and MS/MS fragmentation patterns consistent with database entries. Following the Metabolomics Standards Initiative (MSI) and Schymanski criteria, the compounds reported in this study are assigned Level 2 (putatively annotated compounds based on spectral library matching).

### 4.3. Animals and Experimental Design

Sixty male C57BL/6J mice (8 weeks old, 22–28 g) were obtained from GemPharmatech Co., Ltd. (Nanjing, Jiangsu, China), and housed in a specific pathogen-free facility under controlled conditions (25 ± 2 °C, 55 ± 10% humidity, 12 h light/dark cycle) with free access to food and water. All experiments were approved by the Animal Ethics Committee of Shandong University of Traditional Chinese Medicine (Protocol No. YYLW2024000106) and performed in accordance with the Guide for the Care and Use of Laboratory Animals.

LJF and silybin (Tasly Saint Pharmaceutical Co., Ltd., Tianjin, China) were dissolved in distilled water and administered once daily by oral gavage at 10 mL/kg, with controls receiving vehicle only. The Sil group received 63 mg/kg silybin, while the LJF groups received 0.67, 2.0, and 6.0 g/kg (crude drug equivalent) of LJF (corresponding to 0.31, 0.93, and 2.79 g/kg of freeze-dried extract). The three doses were selected based on the clinical dose range of LJF (6–15 g/day for a 60 kg adult). Using body surface area conversion (mouse/human ratio of 9.1), the clinical equivalent range in mice is approximately 0.91–2.28 g/kg. Thus, the low dose (0.67 g/kg) is slightly below the clinical equivalent, the medium dose (2.0 g/kg) falls within the clinical equivalent range, and the high dose (6.0 g/kg) exceeds the clinical equivalent range, enabling comprehensive dose–response assessment. All doses were confirmed to be safe in preliminary tests. Administration lasted from day 6 to day 15, performed at 08:00 daily, with the liquid diet changed at 17:00. The ALD model was induced in all groups except the Control group, using a chronic-plus-binge ethanol feeding protocol [61]. Briefly, mice were acclimatized to a liquid diet with ethanol concentrations gradually increased from 0% to 4% over the first 5 days. From day 6 to day 15, the EtOH and treatment groups were fed a Lieber–DeCarli liquid diet containing 5% (*v*/*v*) ethanol (Shuyu Biotechnology Co., Ltd., Shanghai, China), while the Control group received an isocaloric liquid diet with maltose-dextrin isocalorically replacing ethanol and was pair-fed to match the volume consumed by the EtOH group on the previous day. On day 16, mice received a single gavage of either 31.5% ethanol or an equal volume of maltose solution (volume (µL) = body weight (g) × 20). Mice were euthanized under isoflurane anesthesia 9 h later, and blood and liver tissues were collected for subsequent analysis.

Sixty mice were randomly assigned to six groups (n = 10/group), and all animals completed the study with no exclusions; the full 10-animal cohort per group was the basis of all analyses. The n = 6 and n = 3 values refer to animals selected from within this cohort for specific assays, not to separate groups. This allocation was predefined, as tissue availability was limited and had to be prioritized for histology. For biochemical assays, the first six pre-assigned ID numbers per group were used; for histology, Western blotting, and transcriptomics, the first three ID numbers per group were used, based on pre-assigned ID numbers in a fixed order rather than formal randomization, with n = 3 predefined according to pilot experiments and the previous literature. Subset selection was independent of outcome data and not based on any phenotypic characteristics. No animals or samples were excluded, and no data points were removed. Blinding was not applied, and group allocation was known to the investigators; however, the primary endpoints were quantitative and instrument-based, which are less susceptible to observer bias than subjective scoring.

### 4.4. H&E Staining and Oil Red O Staining

For H&E staining, formalin-fixed liver samples were embedded in paraffin, sectioned, and stained with hematoxylin and eosin. Histological changes were observed under a light microscope. For Oil Red O staining, frozen liver sections were fixed with formalin, pretreated with 60% isopropanol, and incubated with Oil Red O solution. Microscopic photographs were then recorded.

Liver sections were stained with H&E and Oil Red O. H&E-stained sections were scored by two independent observers blinded to group allocation using the NAFLD activity score (NAS), comprising steatosis (0–3), lobular inflammation (0–3), and hepatocyte ballooning (0–2). For Oil Red O quantification, the positively stained area was measured using ImageJ (version 1.53q; NIH, Bethesda, MD, USA)with a constant color threshold applied across all images, and the Oil Red O-positive area percentage was calculated as the ratio of stained area to total tissue area.

### 4.5. Biochemical Indicators Testing

Serum levels of aspartate aminotransferase (AST), alanine aminotransferase (ALT), total cholesterol (TC), and triglyceride (TG) were measured using commercial assay kits (Nanjing Jiancheng Bioengineering Institute, Nanjing, China) according to the manufacturer’s instructions. Hepatic levels of superoxide dismutase (SOD), glutathione (GSH), low-density lipoprotein cholesterol (LDL-C), and malondialdehyde (MDA) were measured using commercial assay kits (Nanjing Jiancheng Bioengineering Institute, China) according to the manufacturer’s instructions. Levels of interleukin-1β (IL-1β), interleukin-6 (IL-6), and tumor necrosis factor-α (TNF-α) were measured using ELISA kits (BioLegend, San Diego, CA, USA) following the manufacturer’s protocols. ROS levels were assessed using a commercial detection kit (Sevior, Wuhan, China) following the manufacturer’s instructions.

### 4.6. Network Pharmacology

The chemical constituents of LJF were obtained from the TCMSP database. Candidate bioactive compounds were filtered based on oral bioavailability ≥ 30% and drug-likeness (DL) ≥ 0.18, and further supplemented with mass spectrometry data and literature reports. Potential targets of the active compounds were predicted using the SwissTargetPrediction database with default settings, targeting Homo sapiens, and targets with probability scores > 0 were retained. Duplicates were subsequently removed. ALD-associated targets were retrieved from GeneCards, OMIM, DrugBank, and TTD databases. Overlapping targets between LJF and ALD were identified using Venny 2.1.0. Protein–protein interaction (PPI) networks were constructed using the STRING database (Homo sapiens, minimum confidence 0.4) and visualized in Cytoscape 3.7.1. Topological analysis (degree, betweenness centrality, eigenvector centrality, local average connectivity, network score, and closeness centrality) was performed to identify key targets. Pathway enrichment analysis of overlapping genes was conducted using Metascape (*p* < 0.01), and the top biological processes and signaling pathways were visualized as bubble plots or bar charts using the Weishengxin platform.

### 4.7. Transcriptome Sequencing Analysis

Total RNA was extracted from liver tissues (3 mice per group) using TRIzol reagent (Invitrogen, Carlsbad, CA, USA), and RNA quality was assessed using an Agilent 2100 Bioanalyzer (Agilent Technologies, Santa Clara, CA, USA). mRNA was enriched from total RNA using Oligo dT magnetic beads. After fragmentation, first-strand cDNA was synthesized with random hexamer primers, followed by second-strand cDNA synthesis. The library was then prepared through end repair, A-tailing, adapter ligation, fragment size selection, amplification, and purification. After library construction, initial quantification was carried out with a Qubit 2.0 Fluorometer (Invitrogen, Carlsbad, CA, USA), and the library was diluted to 1.5 ng/μL. The insert size was subsequently examined on an Agilent 2100 Bioanalyzer. Once the insert size met the expected range, the effective concentration of the library was accurately determined by qRT-PCR, and a concentration higher than 1.5 nM was required to ensure library quality. After quality control, libraries with different effective concentrations were pooled according to the desired output data volume and then subjected to Illumina sequencing. Sequencing was performed on the Illumina NovaSeq 6000 (Illumina, Inc., San Diego, CA, USA) platform with a paired-end 150 bp (PE150) configuration. Each sample yielded an average of approximately 6.32 Gb of clean data. Sequencing quality metrics indicated that the percentages of bases with Q20 and Q30 scores exceeded 99.3% and 96.89%.

Based on the alignment positions of reads on the reference genome, the number of reads covering each gene (including newly predicted genes) from the start to the end of the gene region was counted. Reads with a mapping quality below 10, non-paired aligned reads, and reads mapped to multiple genomic regions were filtered out. This analysis was performed using the featureCounts tool in the subread software package. Clean reads were aligned to the mouse reference genome (GRCm39) using HISAT2.

After gene expression quantification, genes with significantly different expression levels among samples under different conditions were identified. First, the raw read counts were normalized, mainly to correct for sequencing depth. Then, a statistical model was applied to calculate the *p*-value for hypothesis testing. Finally, multiple hypothesis testing correction was performed to obtain the FDR value. Differentially expressed genes were identified using DESeq2, with |log2(fold change)| ≥ 1 and adjusted *p*-value ≤ 0.05 set as the thresholds.

### 4.8. Real-Time Quantitative Reverse Transcription Polymerase Chain Reaction (RT-qPCR)

Total RNA was extracted from liver tissues using TRIzol reagent(Invitrogen, Carlsbad, CA, USA). Complementary DNA (cDNA) was synthesized from total RNA using the PrimeScript™ RT Reagent Kit (Takara Bio Inc., Shiga, Japan). RT-qPCR was carried out using TB Green Premix Ex Taq II (Takara) on a QuantStudio 5 Real-Time PCR System (Applied Biosystems, Thermo Fisher Scientific, Waltham, MA, USA). The relative expression of target genes was calculated using the 2^−ΔΔCt^ method with GAPDH as the endogenous control. The primer sequence information is shown in Table 2.

### 4.9. Western Blot Analysis

Liver tissues (~20 mg) were homogenized in 400 μL of RIPA lysis buffer supplemented with protease and phosphatase inhibitors and incubated on ice for 30 min. Lysates were centrifuged at 12,000× *g* for 15 min at 4 °C, and the supernatant was collected. Protein concentration was determined using a BCA protein assay kit. Equal amounts of protein (5 μg per lane) were separated by 10% SDS–PAGE and transferred onto PVDF membranes in precooled transfer buffer. Membranes were blocked with 5% non-fat milk or BSA in TBST for 2 h at room temperature and then incubated overnight at 4 °C with primary antibodies against the following proteins: Nrf2 (Proteintech, Rosemont, IL, USA; Cat# 16396-1-AP), HO-1 (Proteintech, Cat# 10701-1-AP), CYP2E1 (Abcam, Cambridge, UK; Cat# ab28146), IκBα (Cell Signaling Technology, Danvers, MA, USA; Cat# 4812), p-IκBα (Cell Signaling Technology, Cat# 2859), p38 (Cell Signaling Technology, Cat# 8690), p-p38 (Cell Signaling Technology, Cat# 4511), p65 (Cell Signaling Technology, Cat# 8242), p-p65 (Cell Signaling Technology, Cat# 3033), and β-actin (Proteintech, Cat# 66009-1-Ig). After washing with TBST, membranes were incubated with HRP-conjugated secondary antibodies for 1 h at room temperature. Protein bands were visualized using an enhanced chemiluminescence (ECL) substrate and imaged using an automated imaging system. Band intensities were quantified using ImageJ software. For non-phosphorylated proteins, band intensities were normalized to β-actin. For phosphorylated proteins, the band intensities of both the phosphorylated and corresponding total proteins were first normalized to β-actin. The phosphorylation level was then calculated as the ratio of normalized phosphorylated protein to normalized total protein.

### 4.10. Statistical Methods

Data analysis was performed using GraphPad Prism software (version 9.0). All data are presented as the mean ± standard deviation (SD). Normality was assessed using the Shapiro–Wilk test, and homogeneity of variances was assessed using the Brown–Forsythe test. When these assumptions were satisfied, one-way ANOVA followed by Dunnett’s post hoc test was used for multiple comparisons; when the assumption of variance homogeneity was not met, Welch’s ANOVA followed by Dunnett’s T3 post hoc test was used. Body-weight changes over time were analyzed using a repeated-measures two-way ANOVA. *p*-values below or equal to 0.05 were considered statistically significant. In all figures, significance levels are indicated as follows: * *p* < 0.05 and ** *p* < 0.01 (compared with the control group). ^#^ *p* < 0.05 and ^##^ *p* < 0.01 (compared with the EtOH group).

## 5. Conclusions

In summary, this study indicates that LJF confers hepatoprotective effects against ALD in mice, and these effects may be associated with the Nrf2/HO-1 pathway and p38 MAPK/NF-κB inflammatory signaling pathway.

## Figures and Tables

**Table 1:** Tentatively annotated ingredients of LJF by UHPLC–Q Exactive HFX MS.

Name	Formula	RT (Time)	Error (ppm)	*m*/*z*	Adducts
Cryptochlorogenic acid	C1_6_H_18_O_9_	4.64	−1.60	353.09	M-H
Isochlorogenic acid C	C_25_H_24_O_12_	6.10	−1.64	515.12	M-H, 2M-H
5-O-Caffeoylshikimic acid	C_16_H_16_O_8_	2.80	−0.16	353.09	M-H + H2O
Secoxyloganin	C_17_H_24_O_11_	5.36	−0.26	807.26	M-H, 2M-H, M + Cl
1-Caffeoylquinic acid	C_16_H_18_O_9_	4.93	−1.52	353.09	M-H
Chlorogenic acid	C_16_H_18_O_9_	4.53	−0.63	355.10	M + H
Luteolin	C_15_H_10_O_6_	7.21	−1.75	285.04	M-H, M + Cl, 2M-H
Liquidambaric acid	C_30_H_46_O_3_	7.32	−0.12	437.34	M + H-H2O, M + H, M + H2O + H
5-O-Feruloylquinic acid	C_17_H_20_O_9_	5.23	−1.65	367.10	M-H
Astragalin	C_21_H_20_O_11_	5.80	−0.24	449.11	M + H
Rutin	C_27_H_30_O_16_	5.61	−0.61	609.15	M-H
3-O-Coumaroylquinic acid	C_16_H_18_O_8_	5.02	−1.10	337.09	M-H
Diosmetin-7-O-Beta-D-glucopyranoside	C_22_H_22_O_11_	6.31	−0.46	463.12	M + H
Hyperoside	C_21_H_20_O_12_	5.78	−0.35	465.10	M + H
Shanzhiside	C_16_H_24_O_11_	1.52	−1.33	391.12	M-H, 2M-H
Quercetin	C_15_H_10_O_7_	5.78	−0.58	303.05	M + H
Nepetin-7-glucoside	C_22_H_22_O_12_	6.17	−0.14	479.12	M + H
(Z)-Aldosecologanin	C_34_H_46_O_19_	6.17	−0.45	757.26	M-H, M + Cl
Loganin	C_17_H_26_O_10_	1.80	−2.18	390.18	M + NH4-H2O
3-Feruloyl-4-caffeoylquinic acid	C_26_H_26_O_12_	6.55	−1.09	529.13	M-H, M + HCOO, M-H + H2O
Isorhamnetin	C_16_H_12_O_7_	6.17	−0.19	317.07	M + H, M + 2CH3CN + H
Diosmetin	C_16_H_12_O_6_	7.97	−0.28	301.07	M + H
Yadanzioside B	C_32_H_44_O_17_	5.48	0.04	791.26	M + HCOOH + HCOO
Quinic acid	C_7_H_12_O_6_	0.77	−1.37	383.12	2M-H
Acanthoside B	C_28_H_36_O_13_	6.21	−0.94	622.25	M + CH3CN + H
Specneuzhenide	C_31_H_42_O_17_	5.20	0.66	703.25	M-H + H2O
Caffeic acid	C_9_H_8_O_4_	4.65	−1.35	179.03	M-H
p-Coumaric Acid Ethyl Ester	C_11_H_12_O_3_	1.08	−0.22	210.11	M + NH4
Kaempferol	C_15_H_10_O_6_	6.10	−0.53	287.05	M + H
Monotropein	C_16_H_22_O_11_	4.66	−0.51	779.22	2M-H
Veratric acid	C_9_H_10_O_4_	2.43	−1.16	227.06	M + HCOO
Euphroside	C_16_H_24_O_10_	4.20	−1.18	421.13	M + HCOO
Protocatechuic acid	C_7_H_6_O_4_	3.33	−1.24	153.02	M-H
Kaempferol 3-O-rhamninoside	C_33_H_40_O_19_	5.50	−0.73	739.21	M-H
Kaempferol-3-O-rutinoside	C_27_H_30_O_15_	5.91	−1.27	593.15	M-H
Lonicerin	C_27_H_30_O_15_	5.61	−0.78	595.17	M + H
beta-Glucogallin	C_13_H_16_O_10_	1.44	−1.21	331.07	M-H
10-Carboxyloganin	C_17_H_23_O_12_	1.61	5.77	452.16	M + CH3OH + H
1,3,5-Tricaffeoylquinic acid	C_34_H_30_O_15_	6.25	0.05	695.16	M-H + H2O
Apigenin	C_15_H_10_O_5_	7.81	−1.24	269.05	M-H

**Table 2:** Primer Information table.

Gene Name	Sequence (5′-3′)	Number of Bases
GAPDH-F	GGGGTCGTTGATGGCAACA	19
GAPDH-R	AGGTCGGTGTGAACGGATTTG	21
Cyp2e1-F	CCGCATCCAAAGAGAGGCACAC	22
Cyp2e1-R	GCACAGCCAATCAGAAAGGTAGGG	24
Hif1a-F	TCTGTGACTTGGCTGACCGAGAG	23
Hif1a-R	CACTCTGGAGGAGGCTCATCTGG	23
Nos2-F	ACTCAGCCAAGCCCTCACCTAC	22
Nos2-R	TCCAATCTCTGCC-TATCCGTCTCG	24

## Data Availability

The original contributions presented in this study are included in the article material. Further inquiries can be directed to the corresponding authors.

## Abbreviations

ALD	Alcohol-associated liver disease
LJF	*Lonicerae japonicae* flos
TC	Total cholesterol
TG	Triglyceride
LDL-C	Low-density lipoprotein cholesterol
ROS	Reactive oxygen species
MDA	Malondialdehyde
SOD	Superoxide dismutase
GSH	Glutathione
HE	Hematoxylin–eosin staining
qRT-PCR	Quantitative real-time PCR
ELISA	Enzyme-linked immunosorbent assay
